# The mitochondrial associated endoplasmic reticulum membranes: A platform for the pathogenesis of inflammation‐mediated metabolic diseases

**DOI:** 10.1002/iid3.647

**Published:** 2022-06-06

**Authors:** Sisay T. Degechisa, Yosef T. Dabi, Solomon T. Gizaw

**Affiliations:** ^1^ Department of Medical Biochemistry, School of Medicine College of Health Sciences, Addis Ababa University Addis Ababa Ethiopia; ^2^ Department of Medical Laboratory Sciences College of Medicine and Health Sciences, Arba Minch University Arba Minch Ethiopia; ^3^ Department of Medical Laboratory Science Wollega University Nekemte Ethiopia

**Keywords:** ER‐stress, inflammatory mediated metabolic diseases, MAM, NLRP3‐inflammasome

## Abstract

Mitochondria‐associated endoplasmic reticulum membranes (MAM) are specialized subcellular compartments that are shaped by endoplasmic reticulum (ER) subdomains placed side by side to the outer membrane of mitochondria (OMM) being connected by tethering proteins in mammalian cells. Studies showed that MAM has multiple physiological functions. These include regulation of lipid synthesis and transport, Ca^2+^ transport and signaling, mitochondrial dynamics, apoptosis, autophagy, and formation and activation of an inflammasome. However, alterations of MAM integrity lead to deleterious effects due to an increased generation of mitochondrial reactive oxygen species (ROS) via increased Ca^2+^ transfer from the ER to mitochondria. This, in turn, causes mitochondrial damage and release of mitochondrial components into the cytosol as damage‐associated molecular patterns which rapidly activate MAM‐resident Nod‐like receptor protein‐3 (NLRP3) inflammasome components. This complex induces the release of pro‐inflammatory cytokines that initiate low‐grade chronic inflammation that subsequently causes the development of metabolic diseases. But, the mechanisms of how MAM is involved in the pathogenesis of these diseases are not exhaustively reviewed. Therefore, this review was aimed to highlight the contribution of MAM to a variety of cellular functions and consider its significance pertaining to the pathogenesis of inflammation‐mediated metabolic diseases.

## Graphical abstract

Aberrations of MAM integrity are considered as a cornerstone in the pathogenesis of several inflammation‐mediated metabolic diseases neurodegenerative diseases, CVD, and cancer.

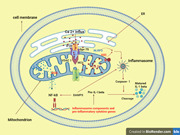

## 1 INTRODUCTION

Subcellular organelles have been viewed as separate entities with defined compositions and organizations that equip them with specialized functions.^1^ However, studies proved that there are interorganellar membrane contact sites in organelles with close tethered proximity.^2^, ^3^ Among such sites, the interaction between the outer membrane of mitochondria (OMM) and that of endoplasmic reticulum (ER) was one of the best characterized.^3^, ^4^

Bernhard et al. came up with the first reported evidence for the existence of sites of physical interaction between ER and OMM from electron microscopic studies of rat liver cells in 1952.^5^ A similar experimental approach by Bernhard and Rouiller in 1956 reassured this finding.^6^ Another study of pseudobranch gland cells of Atlantic killifish in 1959 also reported the existence of this site.^7^ However, the unique membrane that corresponded to this site was isolated as fraction X from a crude rat liver mitochondrial preparation in Vance laboratory in 1990^8^ and later named as mitochondrial‐associated endoplasmic reticulum membranes (MAM) in the paper published in 1994.^9^

**Figure 1:**
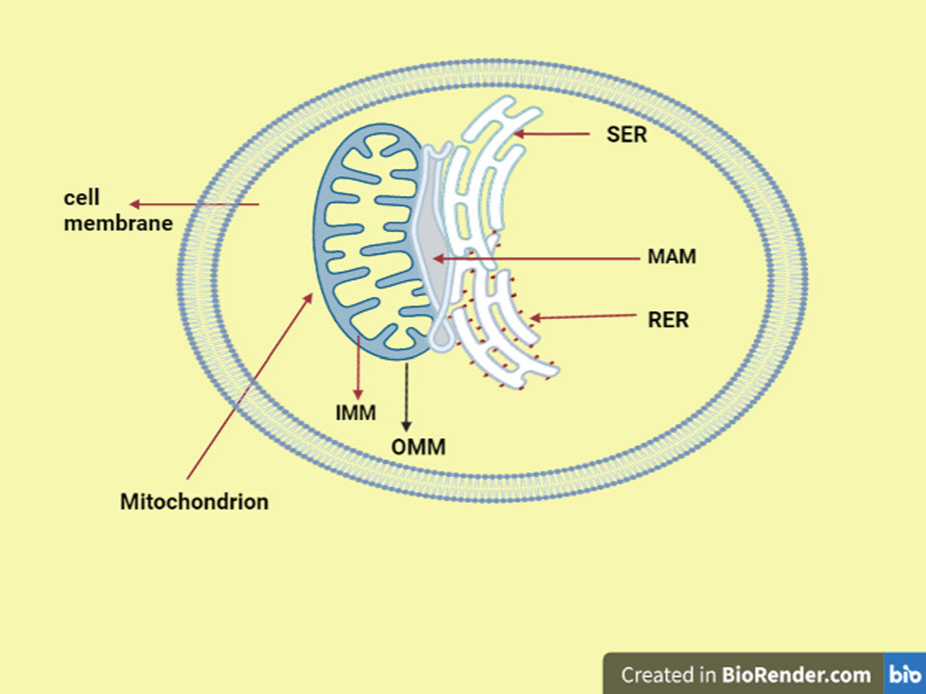
Structure of mitochondrial‐associated endoplasmic membranes. IMM, inner membrane of mitochondrion; MAM, mitochondria associated endoplasmic reticulum membrane; OMM, the outer membrane of mitochondria; RER, rough endoplasmic reticulum; SER, smooth endoplasmic reticulum. The picture is created at https://biorender.com/.

Structurally, MAM is composed of ER subdomains placed side by side with OMM but are biochemically distinct from either pure ER or mitochondria membrane.^10^, ^11^ Electron tomography images revealed that ER and mitochondria are linked by tethers formed from specific protein–protein interactions (Figure 1).^10^

Studies showed that MAM has multiple functions including regulation of lipid synthesis and transport,^8^, ^9^ cellular apoptosis,^11^ initiation of autophagy,^12^ Ca^2+^ transport and signaling,^13^ mitochondrial dynamics,^14^ and insulin signaling.^15^ Most importantly, it serves as a platform for inflammasome formation and activation which play a significant role in initiating inflammatory responses as Thoudam et al.^16^ elegantly explained in their review published in 2018.

Aberrations of MAM integrity were considered as a cornerstone in the pathogenesis of several inflammatory‐mediated metabolic diseases like type 2 diabetes mellitus (T2DM), neurodegenerative diseases including Alzheimer's disease (AD), Parkinson's disease (PD), and amyotrophic lateral sclerosis/frontotemporal dementia (ALS/FTD), cardiovascular diseases (CVD), and cancer.^17^, ^18^, ^19^, ^20^

## 2 STRUCTURE AND COMPOSITION OF MAM

### 2.1 Structure of MAM

When observed under a wide‐field three‐dimensional deconvolution microscope, approximately 5%–20% of the total surface of the mitochondrial network is estimated to be close to the ER membrane.^21^ Moreover, the electron micrograph image revealed that overlapping apposition distances between the ER and OMM vary approximately between 10 and 25 nm.^10^ This variation emanates from the fact that OMM is attached differently to smooth ER and rough ER (Figure 1). The distance of rough ER from OMM is greater than its distance from smooth ER. This is because ribosomes are attached to rough ER and act as spacers, limiting the minimum distance between them to about 20 nm.^22^ The distance can also be varied by the influence of intracellular Ca^2+^ signaling as studies of live cell imaging revealed.^23^, ^24^, ^25^

### 2.2 Composition of MAM

**Figure 2:**
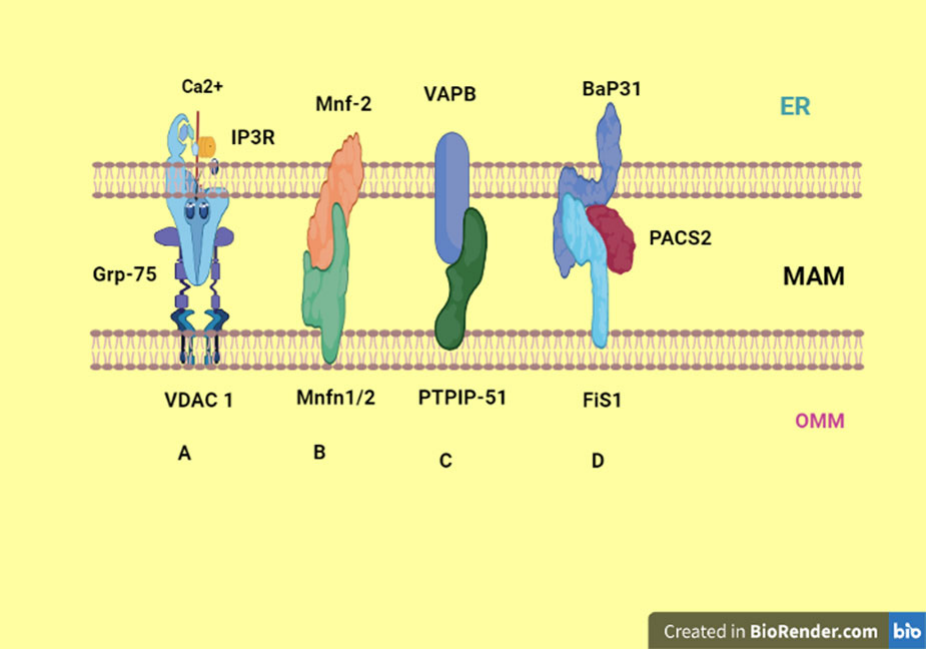
Proposed ER–mitochondria tethering protein complexes. (A) IP3R and VDAC1 interact via GRP75. (B) ER‐located Mfn‐2 interacts with mitochondrial Mfn1/2. (C) VAPB binds to PTPIP51. (D) Bap31 binds to Fis1 and their binding is stabilized by PACS2. The picture is created at https://biorender.com/.

Biochemical methods like subcellular fractionation on a percoll gradient or microscopic techniques like fluorescence microscopy were used for the analysis of the composition MAM fraction.^23^ When the fraction was subjected to proteolysis with trypsin or proteinase K, it detached from mitochondria.^10^ Further analysis using the mass spectroscopy identified more than 1000 MAM resident proteins with variable functions.^26^ Of these proteins, some are involved in tethering the two organelles. MAM tethering proteins form paired complexes (Figure 2). These include Mitofusin‐2 (Mfn2)‐Mitofusin‐1/2 (Mfn1/2), inositol‐1,4,5‐trisphosphate receptor (IP_3_R3)‐glucose‐regulated protein‐75 (Grp75)‐voltage‐dependent anion channel 1 (VDAC1), vesicle‐associated membrane protein‐associated protein B (VAPB)‐protein tyrosine phosphatase‐interacting protein‐51 (PTPIP51), and Fission 1 (Fis1)‐B cell‐associated protein 31 (Bap31).

ER‐located Mfn‐2 interacts in trans with mitochondrial mitofusins to form a tethering complex to bridge the ER and mitochondria and allow efficient Ca^2+^ transfer between them.^27^ Silencing of Mfn‐2 in embryonic fibroblasts has been shown to increase the distance between them Furthermore, the absence of Mfn‐2 consistently cause a loosening of their connection.^18^

The VDAC1 of the OMM interacts with the ER‐Ca^2+^ release channel IP_3_R3 via the molecular chaperone Grp75 and forms the VDAC1–Grp75–IP_3_R3 complex serving as a conduit of Ca^2+^ transfer from the ER to mitochondria. It may not have a tethering role, but rather a contact site spacing/filling function. Sigma1R (Sig‐1R), another MAM resident protein, stabilizes MAM by interacting with VDAC1 and IP_3_R3.^27^, ^28^

VAPB interacts with OMM protein tyrosine phosphatase‐interacting protein‐51 (PTPIP51) and forms the VAPB–PTPIP51 tethering complex.^28^, ^29^ Overexpression of either protein increase ER–mitochondria tethering and Ca^2+^ exchange between them, while their knockout decrease it.^28^, ^30^

Bap31 interacts with Fis1 and forms the Bap31–Fis1 MAM complex.^31^, ^32^, ^33^ Simmen et al. demonstrated that another protein called phosphofurin acidic cluster sorting protein 2 (PACS‐2) modulates the role of Bap31 in tethering the two organelles. However, depletion of PACS‐2 was reported to cause Bap‐31‐dependent mitochondrial fragmentation and uncoupling from the ER along with inhibition of Ca^2+^signal transmission.^34^ Mammalian target of rapamycin complex 2 (mTORC2) also regulates the integrity of MAM by Akt‐dependent phosphorylation of PACS‐2.^35^

### 2.3 Functions of MAM

The existence of contact sites between mitochondria and ER suggests that the structures that are localized to these two different organelles can come together and synergize to provide additional functions at these specialized domains called MAM.^18^, ^27^, ^36^, ^37^

#### 2.3.1 MAM and Ca^2+^ signaling

Ca^2+^ is released from the ER and transferred to mitochondria using MAM as a conduit.^21^ Moderate loading of mitochondria with Ca^2+^ stimulates ATP production via Ca^2+^‐dependent activation of the key metabolic enzymes such as pyruvate dehydrogenase (PDH), isocitrate dehydrogenase, and α‐ketoglutarate dehydrogenase.^27^ However, prolonged overflow of Ca^2+^ into mitochondria activates apoptosis whereas its reduction cellular causes energy crisis by decreasing oxidative phosphorylation.^22^, ^27^

The mechanism of Ca^2+^ transfer from ER to mitochondria is mediated by four major proteins which include IP_3_R, VDAC1, Grp75, and mitochondrial Ca^2+^ uniporter (MCU) reside in MAM, OMM, cytosol, and inner mitochondrial membrane (IMM), respectively.^38^ The VDAC1 of the OMM interacts with IP_3_R via Grp75 (ref. ^27^) and increases the efficiency of mitochondrial Ca^2+^ uptake.^39^ Although OMM is permeable to Ca^2+^ through VDAC1, the IMM is not. Thus, Ca^2+^ needs to go through MCU, a low‐affinity Ca^2+^ channel that requires high Ca^2+^ levels, to reach the mitochondrial matrix.^27^, ^39^, ^40^

Sig‐1R and glucose‐regulated protein 78 (GRP78) are also involved in this process. Sig‐1R physically associate with GRP78 at MAM where they regulate Ca^2+^ flux via IP3R3, stabilizing it and prolonging Ca^2+^ signaling from the ER to mitochondria.^23^ Other proteins also take part in this process. For instance, Akt phosphorylates IP_3_R and suppresses IP_3_R‐mediated Ca^2+^ release, while tumor suppressors phosphatase and tensin homolog (PTEN) directly dephosphorylates IP_3_R and promyelocytic leukemia protein (PML) indirectly dephosphorylates IPR_3_ via sequestration of protein phosphatase 2A (PP2A).^20^

#### 2.3.2 MAM and lipid synthesis and transfer

Phospholipid transport and synthesis is the first recognized function of MAM.^9^ The ER is the main site of phospholipid biosynthesis and plays a significant role in intracellular vesicular trafficking. Because mitochondria are not connected to this trafficking, they require direct lipid transfer from the ER^18^ or they might utilize MAM as a conduit.^24^ On top of this, MAM are also enriched in major enzymes that are involved in the biosynthesis of the two most abundant phospholipids namely phosphatidylcholine  and phosphatidylethanolamine. These enzymes include phosphatidylserine synthase‐1 or ‐2 and phosphatidylethanolamine *N*‐methyltransferase 2.^9^, ^41^, ^42^

Studies reported that MAM is also the site of triacylglycerol synthesis and steroidogenesis.^43^ Long‐chain‐fatty‐acid‐CoA ligase 4 that mediates the ligation of fatty acids to coenzyme A also enriched at MAM.^42^ An enzyme catalyzing the formation of cholesterol esters and diacylglycerol, Acyl‐coenzyme A: cholesterol acyltransferase‐1, is also found in MAM.^42^, ^44^ A MAM resident steroidogenic acute regulatory protein interacts with VDAC2, another MAM protein, and facilitates its translocation to the MAM before it is targeted to mitochondria for its role in steroidgenesis.^45^

#### 2.3.3 MAM and insulin signaling

MAM is also involved in the insulin signaling pathway.^10^ However, several proteins involved in the insulin signaling pathway are enriched in MAM. For example, Akt which phosphorylates IP_3_R and reduces Ca^2+^ release and prevents apoptosis,^10^, ^35^, ^46^ mTORC2 which maintains MAM integrity,^35^, ^47^ PTEN which sensitizes cells to apoptosis by dephosphorylating IP_3_R and restoring Ca^2+^ release^48^ are localized in MAM. PML which modulates its sensitivity to apoptosis by sequestering PP2A and blocking Akt phosphorylation and Ca^2+^ release by IP3R is also found in this site.^49^ Likewise, mitochondrial Ca^2+^ uptake was found crucial for effective insulin signaling in skeletal muscle cells^50^ and cardiac myocytes.^51^

#### 2.3.4 MAM and mitochondrial dynamics

Under normal conditions, mitochondrion changes morphology to create a fragmented or tubular network and to move along the cytoskeleton with coordinated mitochondrial fission and fusion processes.^15^ Mitochondrial fusion assists cells to recover from stressful conditions whereas fission promotes mitophagy to remove mitochondria that are damaged or unable to regain their function to undergo apoptosis.^17^

Mfn‐1, Mfn‐2, and optic atrophy 1 (Opa1) are the most studied of several proteins known to involve in mitochondrial fusion. Mfn‐1 exclusively localizes to mitochondria, whereas Mfn‐2 resides in MAM and mitochondria. While Mfn‐1 and Mfn2 are responsible for the fusion of the OMM, Opa1 is responsible for the fusion of the IMM.^15^ Likewise, the fission process also involves several proteins of which dynamin‐related protein 1 (Drp1) is well studied and recruited from the cytosol to the OMM by various adaptor proteins including mitochondrial fission protein 1 (Fis‐1) which are present on the OMM. DRP1 is translocated to the MAM site, where it can cleave mitochondria efficiently and target damaged mitochondria for mitophagy.^15^, ^20^

#### 2.3.5 MAM and autophagy

“Autophagy is a mechanism for the degradation of cellular material either as a way to provide nutrients during times of starvation or as a quality control system that eliminates unneeded proteins and/or organelles during normal growth and development”^13^ These wastes are isolated by double‐membrane vesicles called autophagosomes which fuse with lysosomes to form autolysosomes and eventually degraded by lysosomal enzymes.^52^ The formation and development of autophagosomes involve autophagy‐related genes, which encode proteins that regulate autophagy as discussed in various articles.^53^, ^54^, ^55^, ^56^

Hamasaki et al.^54^ reviewed that the origin and formation of autophagosomes remain obscure for scientists though independent studies have pointed to several different organelles as potential membrane sources. However, a recent study showed that autophagosomes form at MAM.^13^

Gomez‐Suaga et al. reported that VAPB‐PTPIP51 tethers are also in regulating autophagy. However, overexpression of VAPB or PTPIP51 tightens ER–mitochondria contacts and impairs autophagosome formation. However, small interfering RNA (siRNA)‐mediated loss of VAPB or PTPIP51 loosens contacts and stimulates autophagosome formation.^57^

#### 2.3.6 MAM and cellular apoptosis

The transfer of Ca^2+^ from ER to mitochondria is accomplished by MAM and excessive mitochondrial Ca^2+^ uptake can trigger Ca^2+^‐mediated apoptosis.^58^ Higher matrix Ca^2+^ levels sensitize mitochondria to undergo mitochondrial outer membrane permeabilization (MOMP), a process preceding apoptosis.^59^ Increased uptake of Ca^2+^ by mitochondria may result in changes in the permeability of the IMM. This is caused by the prolonged opening of the mitochondrial permeability transition pore which induces mitochondrial swelling and OMM rupture. This is followed by the release of apoptosis‐inducing caspase‐activating factors such as cytosolic cytochrome *C*.^58^ The released cytochrome *C* amplifies caspase activation by binding to the IP_3_R and exacerbating its Ca^2+^ leaking properties.^60^

Bap31–Fis1 complex also play role in apoptosis by recruiting caspase‐8 which enables the cleavage of Bap31 into its pro‐death fragment, p20Bap31. This fragment favors the emptying of ER–Ca^2+^ stores and induces cell death.^33^ Moreover, PTEN has been known to interact with IP3R/Akt complex and reduce their phosphorylation. This, in turn, results in increased Ca^2+^ release and apoptosis.^48^

#### 2.3.7 MAM and ER stress

“The ER plays an indispensable role in protein folding. This role is facilitated by the presence of chaperone proteins capable of binding to newly synthesized, but as yet unfolded, proteins to facilitate optimal protein folding and prevent protein‐protein aggregation under normal physiological conditions.”^61^ However, in pathological conditions, the accumulation of misfolded or unfolded proteins may occur and cause cellular dyshomeostasis. This triggers ER to elicit an adaptive or protective response called unfolded protein response (UPR) which restores cellular homeostasis.^60^, ^61^ If the homeostasis is not restored, the UPR switches to promote apoptosis. Nevertheless, in some pathophysiological situations, the homeostatic capacity of the ER and the UPR may not meet cellular demands and may even become a detrimental condition called ER stress in which structural uncoupling of ER from mitochondria may also induce it.^61^, ^62^, ^63^

#### 2.3.8 MAM in the formation and activation of the NLRP3 inflammasome

Cells require the capacity to sense and respond to the danger presented by extrinsic threats. Pattern recognition receptors (PRRs) recognize conserved molecular patterns expressed by invading pathogens (pathogen‐associated molecular patterns, PAMPs) or endogenous ligands derived from cellular damage resulting from infection or tissue injury (danger‐associated molecular patterns, DAMPs). Activation of PRRs by PAMPs or DAMPs triggers downstream signaling cascades and causes the production of Type I interferon (interferon‐α and interferon‐β) and pro‐inflammatory cytokines resulting in inflammation. DAMP‐triggered inflammation was reported to play a crucial role in the pathogenesis of inflammation‐mediated metabolic diseases.^61^

One of the innate immunity sensors that mediate this inflammatory response are cytosolic multiprotein complexes termed inflammasomes^23^ and the formation of this inflammasome involves MAM as a platform.^60^, ^65^ The most studied inflammasome was the NOD‐like receptor family protein 3 (NLRP3) inflammasome.^65^

**Figure 3:**
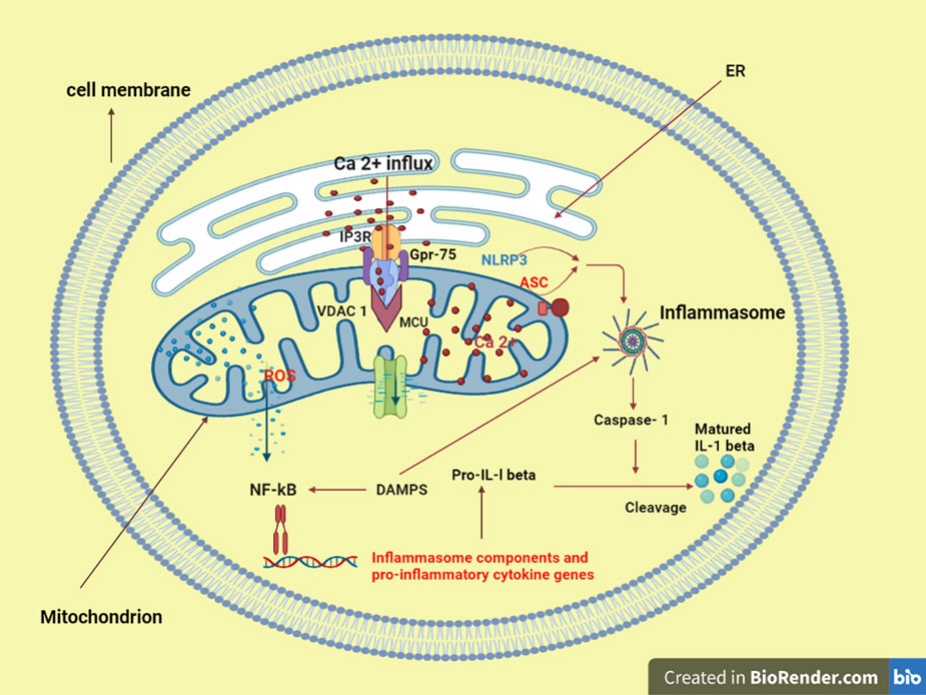
MAM are important sites for NLRP3‐inflammasome formation and activation (described in Section 2.3.8). The picture is created at https://biorender.com/.

In an inactive state, NLRP3 localizes to the ER membrane and cytosol. However, in its active state, both NLRP3 and its adaptor apoptosis‐associated speck‐like protein containing a CARD (ASC) relocate to the MAM fraction where they are strategically assembled and located to sense signals emanating from mitochondria like increased ROS and mitochondrial‐derived DAMPs like mitochondrial DNA (mtDNA), ATP, cardiolipin, cytochrome *C*, and succinate.^19^ NLRP3 oligomerizes and exposes its effector domain to interact with ASC. ASC in turn recruits pro‐caspase‐1 which is cleaved and becomes matured caspase‐1. Finally, activated caspase‐1 cleaves pro‐interleukin‐1β (pro‐IL‐1β) and pro‐IL‐18 to generate mature IL‐1β and IL‐18 (Figure 3).^20^

Recently, Zang et al. reported that the NLRP3 inflammasome has been shown to be activated by a variety of distinct stimuli, including K+ efflux, mitochondrial dysfunction, lysosomal disruption, and trans‐Golgi disassembly. However, the most widely accepted stimuli was K+ efflux‐induced NLRP3 inflammasome activation. This mechanism has been thought to involve mitochondria. This is supported by the fact that PAMPs such as bacterial lipopolysaccharide (LPS) induced the expression of genes involved in mitochondrial biogenesis and mitophagy, resulting in an increase in mitochondrial mass and mitochondrial membrane potential. To back up their claim, the researchers silenced the mitochondrial transcription factor A (Tfam), and genetic ablation of Tfam abolished the NLRP3 inflammasome activation induced by K^+^ efflux via release of mtDNA as deprivation of cellular mtDNA by ethidium bromide treatment could reverse inflammasome activation induced by K+ efflux. They also revealed that mtDNA release induced by K+ efflux in macrophages activates NLRP3 inflammasome.^66^

It has also been shown that ER stress activates the NLRP3 inflammasome in both peripheral and central immune cells. ER stress‐induced NLRP3 inflammasome activation occurs via a Ca^2+^‐dependent and ROS‐independent mechanism in monocytes, which is associated with upregulation of MAMs‐resident chaperones, closer ER–mitochondrial contacts, mitochondrial depolarization, and impaired dynamics. MAM thus plays an important role in the innate immune cells' response to ER stress.^67^

## 3 MAM AND PATHOGENESIS OF INFLAMMATION‐MEDIATED DISEASES

### 3.1 Neurodegenerative diseases

Neurodegenerative diseases, such as AD, PD, and ALS/FTD, occur when nerve cells in the brain or peripheral nervous system lose function over time and ultimately die.^68^ While they involve distinct protein pathologies, they share similar features that involve MAM disruption including mitochondrial damage, Ca^2+^ homeostasis, lipid metabolism, axonal transport, UPR activation, autophagy, and inflammatory responses.^36^

Inflammatory response proteins have been most commonly implicated in neurodegenerative diseases. For example, a continuous release of IL‐1β negatively modulates the integrity of the brain–blood barrier, which results in the infiltration of immune cells into the central nervous system.^19^ The same cytokine amplifies the generation of other pro‐inflammatory factors by stimulating the activation of microglia and astrocytes.^69^ Moreover, Fogal et al.^70^ have demonstrated that overexpression of IL‐1β mediates neuronal injury and cell death throughout glutamate excitotoxicity.

Misfolded protein aggregates and excessive accumulation of metabolites are also critical determinants for the activation of ER‐stress and NLRP3 inflammasome which in turn initiates neurodegeneration including AD and PD.^64^

#### 3.1.1 Alzheimer's disease

The pathogenesis of AD and the series of events underlying it are unknown. The most widely accepted hypothesis is called the amyloid cascade, based on the observation that the brain of AD patients contains high levels of extracellular plaques called β‐amyloid (Aβ) composed of 40–42 amino acids and neurofibrillary tangles (NFTs) which are composed of hyperphosphorylated forms of the microtubule‐associated protein tau in the cerebrum. Aβ is produced by cleavage of the amyloid precursor protein (APP) by presenilin (presenilin‐1 and/or presenilin‐2), both of which are active components of the γ‐secretase complex.^69^ Notably, dominant mutations both in the presenilins and in APP are currently the only known causes of the familial form of AD (FAD).^71^, ^72^ As summarized in references^17^, ^73^ these two isoenzymes of presenilins were found to be enriched in MAM fractions from neuronal and non‐neuronal cells. Yu et al.^74^ briefly explained that significant mutations in APP or/and PSEN1/2 might lead to the excessive generation of Aβ42 and the increased ratio of Aβ42/40 which result in AD in their recent review.

AD that is linked with presenilin mutation is also characterized by increased levels of monocyte chemoattractant protein 1 (MCP‐1), IL‐6, and IL‐8 while a Presenilin1 mutation in microglial cells amplified tumor necrosis factor α, IL‐1α, IL‐1β, and IL‐6 gene expression.^72^ It was also reported that APP and its catabolites are also found in MAM, where they interact with other MAM‐resident proteins and modulate ER functions.^75^

The relationship between MAM and NLRP3 inflammasome is already described in Section 2.3.8. Moreover, researchers reported the intimate relationship between amyloid‐β and NLRP3 inflammasome as oligomerized Aβ originating from nontoxic Aβ monomers directly interacted with NLRP3, leading to the activation of the NLRP3 inflammasome.^76^, ^77^ Heneka et al. demonstrated that the deposition of Aβ drives cerebral neuro‐inflammation by activating microglia. Indeed, Aβ activation of the NLRP3 inflammasome in microglia is fundamental for IL‐1β maturation. The researchers explained their claim with a piece of evidence from NLRP3−/− or caspase‐1−/− mice carrying mutations associated with familial AD. These mice, which were largely protected from loss of spatial memory, demonstrated reduced brain caspase‐1 and IL‐1β activation, enhanced Aβ clearance, and NLRP3 inflammasome deficiency skewed microglial cells to an M_2_ phenotype and resulted in the decreased deposition of Aβ.^78^

#### 3.1.2 Parkinson's disease

PD is the most common movement disorder and the second most common neurodegenerative disease after AD.^23^, ^36^ It is characterized by an excessive death of dopaminergic neurons in the substantia nigra pars compacta together with intraneuronal inclusions termed “Lewy bodies” which are mainly formed from aggregates of a protein called α‐synuclein. Most recently, it has been shown that α‐synuclein localizes at the MAM.^36^, ^75^ The overexpression of both wild type and mutant α‐synuclein isoforms disrupt the VAPB‐PTPIP51 tethers, thus decreasing MAM formation. This causes decreases in Ca^2+^ exchanges between the two organelles that, in turn, lowers mitochondrial ATP production.^28^

Additionally, pathogenic mutations of α‐synuclein causes downregulation of MAM functions while activating inflammasome. Indeed, α‐synuclein aggregates were found to be sufficient to provoke IL‐1β production by activating microglia and astrocytes. The fibrillary and monomeric forms of this protein showed differences in their capacity to induce inflammation. The monomeric form only induces the expression of pro‐IL‐1β whereas the fibrillary form can provoke caspase‐1 activation and maturation of IL‐1β and fully activates the inflammasome.^79^, ^80^

In fact, similar to AD, stimulating caspase‐1 activation and the release of IL‐1β is necessary to induce the production of ROS and activity of cathepsin‐B.^81^ Accordingly, through specific inhibition of cathepsin‐B; it is possible to interfere with the inflammasome assembly though this finding was not validated.

More notably, Yan et al. showed that dopamine‐producing neurons and NLRP3 inflammasome are tightly interconnected and are able to regulate each other. They further showed that the neurotransmitter dopamine has the potential to inhibit NLRP3 inflammasome activation and subsequent IL‐1β production. This inhibitory activity of dopamine occurs via the dopamine D1 receptor signaling through an autophagic dependent process.^82^

#### 3.1.3 ALS with associated front temporal dementia

ALS/FTD is a neurodegenerative disease caused by the loss of motor neurons, resulting in the gradual deterioration of muscles. The exact cause of ALS is still not clear. However, a mutation in SigR1 is discovered in a juvenile form of ALS. In the SigR1 knockout mouse, ALS phenotypes such as muscle weakness and motor neuron loss were exhibited.^27^

Another MAM protein, VAPB is also mutated in familial ALS. A mutant VAPB increases its affinity to PTPIP51 and strengthens VAPB‐PTPIP51 tethering, which alters Ca^2+^ shuttling between ER and mitochondria as elegantly summarized in a recently published review by Lee and Min.^83^ Dominantly inherited forms of the disease were caused by deposits of Tar DNA‐binding protein 43 (TDP‐43) gene mutation. Notably, TDP‐43‐induce alteration of MAM involves breaking of the VAPB–PTPIP51, causing aberrant cellular Ca^2+^ homeostasis and decreased rates of ATP production.^36^

Expression, activation, and co‐localization of the NLRP3 inflammasome were observed in the spinal cord of male SOD1 (G93A) mice carrying a mutant human superoxide dismutase 1 (SOD1).^84^ It was also demonstrated that both aggregated and soluble SOD1G93A activates the inflammasome in primary mouse microglia.^85^ However, SOD1G93A was unable to induce IL‐1β secretion from microglia pretreated with NLPR3 or deficient for NLRP3,r, confirming NLRP3 as the key inflammasome complex mediating SOD1‐induced microglial IL‐1β secretion.^86^ Microglial NLRP3 upregulation was also observed in the TDP‐43 mutant mice model. TDP‐43 could also activate microglial inflammasomes in an NLRP3‐dependent manner. Mechanistically, they could identify the generation of ROS and ATP as key events required for SOD1G93A‐mediated NLRP3 activation.^84^, ^87^

### 3.2 Diabetic mellitus

Insulin resistance and pancreatic β‐cell dysfunction in T2DM are widely associated with derangement of MAM compartments as there is a strong linkage between MAM integrity and insulin action in hepatic cells.^88^ It was also demonstrated in vitro and in vivo that defective MAM is closely associated with impaired hepatic insulin sensitivity and restoration of MAM integrity by cyclophilin D overexpression improved insulin signaling in primary hepatocytes of diabetic mice.^89^

Notably, an in vivo experimental study reported that in the skeletal muscle of obese and diabetic humans, the expression levels of the ER–mitochondria tethering protein Mfn‐2 are reduced. Indeed, the livers of transgenic mice deleted for Mfn‐2 possessed a low insulin response and a reduction in mitochondrial respiration resulting in increased production of ROS which cause subsequent accumulation of mutation at the level of mtDNA.^86^ An increase in the level of ROS was found to be a primary contributor to inflammation in T2DM. In fact, pro‐inflammatory cytokines also exacerbate ER and oxidative stress events, leading to β‐cell loss, recruitment of NLRP3 inflammasome, and finally the pathogenesis of T2DM. Moreover, increased ROS also stimulates conformational changes in thioredoxin‐interacting protein (TXNIP) and subsequent loss of the complex thyroidotoxin (TRX)–TRXNIP that binds and activates NLRP3 resulting in the generation of IL‐1β.^23^

### 3.3 Cardiovascular diseases

#### 3.3.1 Mitochondrial dynamics and cardiovascular disease

MAM and mitochondrial dynamics are also recognized as key factors in the pathogenesis of CVD. This was evidenced by a study that demonstrated that precise Ca^2+^ transport from the ER to the mitochondria regulates the cardiac contraction cycle.^90^ Moreover, mitochondrial Ca^2+^ fluctuations and Ca^2+^ oscillation triggered by ER are present during cardiomyocyte beating.^91^

Among the proteins involved in the maintenance of MAM, Mfn1/2 seems to be the most relevant one in the pathogenesis of CVD. It was confirmed that adult hearts deleted for both mitofusins showed compromised cardiac function, augmented left ventricular end‐diastolic volume, and reduced fractional shortening. This is supported by the fact that transgenic Mfn‐2−/− mice exhibited reduced contact length between these organelles resulting in a reduction of ER–mitochondrial Ca^2+^ transfer, and increased production of ROS that activate the NLRP3 inflammasome.^85^

Finally, it has been reported that specific proteins conserving the ER–mitochondria interface are involved in ischemia/reperfusion (I/R). For example, OPA1 deficiency was associated with increased sensitivity to I/R, whereas the inhibition of Fis1 and DRP1 function was reported to be cardioprotective.^92^

#### 3.3.2 MAM and cardiovascular diseases

Missiroli et al. briefly summarized in their review that excess ROS production and subsequent NLRP3 activation are frequently found in CVD. In the presence of excess cholesterol deposition in the arterial wall, it forms crystals that induce inflammatory injury. This can be supported by the finding that macrophages can internalize these crystals and promote NLRP3 inflammasome activation in a process involving leakage of cathepsin B and L into the cytoplasm. This, in turn, causes the excessive formation of mitochondrial ROS and lowering in potassium concentrations.^19^

The important role of inflammasomes was confirmed in atherosclerosis using ApoE−/− mice deletion of IL‐1β gene reduced the size of atherosclerotic lesions by up to 30%.^93^ Moreover, the deletion of the IL‐ 18 receptors (IL‐18R−/−) decreased the size of the lesions.^94^ Despite this, NLRP3 may not be the only source of pro‐inflammatory cytokines in atherosclerosis. Transgenic mice ApoE−/− crossed with mice deleted for different components of the NLRP3 such as (Nlrp3−/−, ASC−/−, or caspase‐1−/−) exhibited no differences in atherosclerotic lesions and plaques when compared to the double knockout and control mice.^95^

NLRP3 inflammasome recruitment and the appropriate MAM composition also have an important role during I/R. Notably, IL‐1β and IL‐18 are primary mediators of I/R‐induced human myocardial injury through the inhibition of caspase‐1 activity that reduces the depression in contractile force after I/R.^96^ Similarly, in ASC−/− mice the level of inflammatory cytokines was reduced and this results in a significant reduction of injuries such as the development of infarctions, myocardial fibrosis, and dysfunction in myocardial I/R injury compared to wild‐type controls.^97^

Additionally, Shengnan and Ming‐Hui demonstrated that FUNDC1 also participates in MAM formation in cardiomyocytes by binding to IP3R2. This is because FUNDC1 deletion causes an 80% reduction in ER and mitochondria contact sites resulting in the decrease of Ca^2+^ transfer from ER to mitochondria resulting in elevation of ROS generation which induces chronic inflammation.^1^

### 3.4 The role of MAM in the onset and progression of cancer

#### 3.4.1 Alteration of MAM composition in breast cancer

In breast cancers, the expression of the stress‐activated Sig1R was found to be higher in metastatic potential cancer cells than in normal tissue. Under basal conditions, Sig1R binds the MAM chaperone GRP78; however, upon activation of IP_3_R3, Sig1R dissociates from GRP78 and binds the receptor, thereby stabilizing it at the MAM and enhancing IP_3_R3‐mediated Ca^2+^ fluxes to the mitochondria. However, during conditions of chronic ER stress involving prolonged ER Ca^2+^ depletion, Sig1R translocates from MAM to the peripheral ER and attenuates cellular damage, thereby preventing cell death. Sig1R also regulates Ca^2+^ homeostasis by forming a functional molecular platform with the calcium‐activated K^+^ channels, thus driving Ca^2+^ influx and favoring the migration of cancer cells. This implicates protumorigenic functions of this protein as stated in a current review by Morciano et al.^11^

#### 3.4.2 Alteration of MAM in hepatocellular cancer

Alteration of Mnfs or OPA1 function leads to decreased mitochondrial fusion, shifting the balance of mitochondrial dynamics to over‐fragmentation. This phenomenon was observed in experimental settings, aimed to investigate cancer biology.^57^ For instance, a study demonstrated that MFN1 loss‐of‐function triggered the epithelial‐to‐mesenchymal transition of hepatocellular carcinoma favoring its metastasis.^98^ Another study demonstrated that knockdown of Mfn‐1 and OPA1 inhibited mitochondrial fusion in experimental settings, leading to reduced cell growth and tumor formation. This implicates the antitumor effect of OPA1 and Mfn‐1 by silencing the induction of proapoptotic mechanisms, inhibition of oxidative metabolism, and ATP production.^99^

## 4 CONCLUSION

MAM, a tiny membrane contact site, serves a far more important physiological function than most people realize. Based on the physiological function of multiple MAM resident proteins, there are still many unanswered questions about these contact sites. Apparently, Ca^2+^ homeostasis, mitochondrial dynamics, inflammasome formation and activation, cellular autophagy, and apoptosis are all affected when this membrane contact site is disrupted. The cumulative effect of its disruption is strongly associated with inflammatory‐mediated metabolic diseases, and it has a dramatic impact on health. MAM, on the other hand, plays an important role in innate immune cell response to ER stress and serves as a site of NLRP3 inflammasome activation under stress conditions, implying that MAM could serve as a novel potential therapeutic target for inflammatory‐related metabolic diseases. However, the nonspecific alteration of MAM makes it so difficult to use it as a target to treat some of these diseases.

## AUTHOR CONTRIBUTIONS

Sisay Teka Degechisa wrote the manuscript draft. Yosef Tsegaye Dabi and Solomon Tebeje Gizaw contributed to the gathering of data, draft reviewing, and editing of the manuscript. All authors revised the manuscript and approved the final version of the manuscript before submission.

## CONFLICT OF INTEREST

The authors declare no conflict of interest.

## Data Availability

Data sharing is not applicable to this article as no data sets were generated or analyzed during the current study.
